# Editorial: Neuroglial antibodies: From clinical associations to pathophysiological investigations

**DOI:** 10.3389/fneur.2023.1143410

**Published:** 2023-02-01

**Authors:** Alberto Vogrig, Cristina Valencia-Sanchez, Jérôme Honnorat, Sergio Muñiz-Castrillo

**Affiliations:** ^1^Clinical Neurology, Udine University Hospital, Azienda Sanitaria Universitaria Friuli Centrale (ASU FC), Udine, Italy; ^2^Department of Medical Area (DAME), University of Udine, Udine, Italy; ^3^Department of Neurology, Mayo Clinic Arizona, Scottsdale, AZ, United States; ^4^French Reference Center for Paraneoplastic Neurological Syndromes and Autoimmune Encephalitis, Hôpital Neurologique, Hospices Civils de Lyon, Bron, France; ^5^Team SynatAc, MeLiS Institute-UCBL-CNRS UMR 5284-INSERM U1314, Université Claude Bernard Lyon 1, Lyon, France; ^6^Center for Sleep Sciences and Medicine, Stanford University, Palo Alto, CA, United States

**Keywords:** autoimmune encephalitis, paraneoplastic neurological syndromes, MOGAD, NMOSD, autoimmune epilepsy

The aim of the present Research Topic is to provide a cutting-edge overview of neurological autoimmune disorders with neuroglial antibodies, focusing on distinctive clinical presentations, previously neglected aspects, evaluations of tests for antibody detection, new serum and/or cerebrospinal fluid (CSF) biomarkers, treatment, and outcome. A total of seven articles are collected here: five original studies as well as two comprehensive review papers.

The review paper by Fernández-Fournier et al. aims to guide clinicians into diagnostic tests currently adopted for the study of neuroglial antibodies, including possible indications, limitations, and potential pitfalls. This work highlights several aspects that are relevant in clinical practice, which were stressed in the recently updated diagnostic criteria for paraneoplastic neurological syndromes (PNS) (1): (i) the finding of neural antibodies should always be considered in the context of the patient's clinical presentation, as indiscriminate testing increases the chances of both false-positive and false-negative results; (ii) commercial kits testing multiple antibodies are helpful, but the diagnostic yield is low if no confirmatory tests are used, particularly for some antibodies assessed by line blots (2); (iii) ideally, CSF and serum should be tested in parallel. These aspects are particularly relevant for rare diseases such as PNS, autoimmune encephalitis (AE), neuromyelitis optica spectrum disorders (NMOSD), and myelin oligodendrocyte glycoprotein antibody-associated disease (MOGAD), in which epidemiological studies recently showed their incidence and prevalence at the population level (3–5). According to Bayes theorem, even superb tests fail in the presence of low-prevalence disorders, so the decision on which patients should be tested for neuroglial antibodies is crucial (6).

The original paper by Bartley et al. highlights the relevance of tissue-based immunofluorescence assays in the diagnosis of suspected autoimmune central nervous system disorders (1), and the role of new techniques to identify novel neural antibodies such as Phage display immunoprecipitation sequencing (PhIP-Seq) (7), which previously led to the discovery of Kelch-like protein 11 IgG (8). The authors describe a patient with HIV who presented with steroid responsive meningoencephalitis and whose CSF analysis showed an unknown neural antibody with immunoreactivity against structures of the axon initial segment and node of Ranvier. The antigen was later identified as ankyrin G by PhIP-Seq. Ankyrin G antibody was then found retrospectively in an additional case, a patient with ovarian cancer and seizures, and not found in controls, indicating that ankyrin G could be a biomarker of neurological autoimmunity.

In addition to antibody testing, other paraclinical tests are important in confirming the diagnosis of AE and deciding the need to escalate immunotherapy. To this regard, Cornacchini et al. explore the usefulness of tools such as 18F-fluoridesoxyglucose-positron emission tomography (18F-FDG-PET) and long-term monitoring video EEG (LTMV EEG) in a patient with anti-leucine-rich glioma-inactivated 1 (LGI1) encephalitis. Following evidence of disease activity acquired using the above-mentioned tests, the decision of treatment escalation with rituximab was made, resulting in clinical improvement of the patient. On similar grounds, Chen et al. examined the potential use of responsive neurostimulation (RNS^®^, NeuroPace) for diagnostic and therapeutic purposes in a patient with antibody-negative but probable autoimmune-associated epilepsy. Despite lack of effect on seizure frequency, the use of electrocorticography (ECoG) data provided an objective surrogate measure of seizure frequency, which can be helpful in assessing treatment response, including the effect of immunotherapy, in this challenging scenario where the effect of antiseizure medications is usually poor.

Regarding treatment approaches for AE, this Research Topic focuses on previously neglected aspects such as the one tackled by Iyengar et al. on the effect of type of surgery (“aggressive” vs. “conservative”) in paraneoplastic cases of anti-N-methyl-D-aspartate receptor (NMDAR) encephalitis associated with ovarian teratoma, finding that aggressive approaches did not improve 1-year functional outcomes. This important result awaits confirmation in prospective studies with larger number of patients, which will permit to avoid potential biases (e.g., “aggressive” approaches are usually reserved for more severe cases).

Two additional studies included in this Research Topic focus on antibody associated inflammatory demyelinating syndromes of the central nervous system. In particular, the review paper by Budhram et al. depicts the novel clinico-radiographic spectrum of unilateral cortical FLAIR-hyperintense Lesions in Anti-MOG-associated Encephalitis with Seizures (the acronym “FLAMES” was coined previously by the same authors) (9). Cerebral cortical encephalitis is a recently described MOGAD phenotype characterized by headache, seizures, and encephalopathy (10). Magnetic resonance imaging findings involve unilateral or less frequently bilateral cortical T2 hyperintensity; leptomeningeal enhancement is common. Interestingly, NNMDAR antibodies can coexist in a minority of cases. Most patients improved with steroids (10). Hence, this review summarizes the clinical presentations and imaging findings reported in the literature of MOGAD patients with predominantly cortical and meningeal involvement, raising awareness of a wider spectrum of clinical manifestations in MOGAD, which may allow for earlier diagnosis and treatment.

Finally, the original paper by Bauer et al. comprehensively investigated serum cytokines and chemokines of MOGAD and NMOSD as compared to multiple sclerosis, shedding light on the pathogenesis of these conditions. Interestingly, patients with MOGAD and NMOSD showed a, partially shared, predominant Th17 profile compared to multiple sclerosis. Furthermore, some upregulated cytokines in pediatric patients with MOGAD might suggest an infectious trigger. Thus, the identification of cytokine and chemokine profiles that differentiate these disorders can aid in diagnosis, development of treatments directed against specific targets (such as the anti-interleukin 6 agent satralizumab for NMOSD), and monitoring response to treatment.

In conclusion, this Research Topic focuses on the most challenging scenarios (rare clinical presentation, diagnostic pitfalls, novel biomarkers, and treatment approaches in refractory cases) in the field of autoimmune neurological disorders with neuroglial antibodies. As Guest Editors, we hope that these manuscripts will inspire our readers to further explore these topics.

## Author contributions

AV, CV-S, JH, and SM-C: study concept, design, and drafting the manuscript. All authors contributed to the article and approved the submitted version.
